# *‘I’m going to look for you and take your kids’*: Reproductive justice in the context of immigration enforcement

**DOI:** 10.1371/journal.pone.0217898

**Published:** 2019-06-04

**Authors:** Paul J. Fleming, William D. Lopez, Charo Ledon, Mikel Llanes, Adreanne Waller, Melanie Harner, Ramiro Martinez, Daniel J. Kruger

**Affiliations:** 1 Department of Health Behavior & Health Education, University of Michigan School of Public Health, Ann Arbor, Michigan, United States of America; 2 Acción Buenos Vecinos, Ypsilanti, Michigan, United States of America; 3 Department of Family Medicine, University of Michigan Medical School, Ann Arbor, Michigan, United States of America; 4 Washtenaw County Public Health, Ypsilanti, Michigan, United States of America; 5 Washtenaw Interfaith Coalition for Immigrant Rights, Ypsilanti, Michigan, United States of America; 6 Population Studies Center, University of Michigan, Ann Arbor, Michigan, United States of America; University of Cape Town, SOUTH AFRICA

## Abstract

Prior research has shown that immigration law enforcement contributes to poor health outcomes—including reproductive health outcomes—among Latinos. Yet no prior research has examined how immigration enforcement might inhibit reproductive justice and limit individual’s reproductive autonomy. We utilized data from an existing study that consisted of a partnership with a Latino community in Michigan in which an immigration raid resulted in multiple arrests and deportations midway through data collection. Using cross-sectional survey data (*n* = 192) where no one was re-interviewed, we used ordinal logistic regression to compare desired pregnancy timing of individuals surveyed prior to and after the raid to determine the impact of an immigration raid on desired timing of next pregnancy. We then used qualitative data—including 21 in-depth interviews and participant observation—collected in the community after the raid to contextualize our findings. Controlling for socio-demographic characteristics, we found that Latinos surveyed in the aftermath of the raid were more likely to report a greater desire to delay childbearing than Latinos surveyed before the raid occurred. Our qualitative data showed that an immigration raid has financial and psychological effects on immigrant families and that a raid may impact reproductive autonomy because people are fearful of these impacts. These finding suggest that current immigration enforcement efforts may influence reproductive decision-making, impede Latinos reproductive autonomy, and that family-friendly immigration policy reform is needed.

## Introduction

The concept of reproductive autonomy refers to “the power to decide when, if at all, to have children.”[1] Women of color in the United States developed the concept of reproductive justice—closely tied to reproductive autonomy—in the 1990s to extend beyond the individualized framing of autonomy and acknowledge the structural factors that constrain autonomy.[2] Ross and colleagues describe the three primary values of reproductive justice: the right to have children, the right to not have children, and the right to parent one’s children in safe and healthy environments.[2]

The concept of reproductive justice represents a significant shift from traditional notions of reproductive rights, specifically in its inclusion of the impact of contextual and structural factors on reproduction. As described by Ross and colleagues, “[T]the ability of any woman to determine her own reproductive destiny is directly linked to the conditions in her community and these conditions are not just a matter of individual choice and access. For example, *a woman cannot make an individual decision about her body if she is part of a community whose human rights as a group are violated*, such as through environmental dangers or insufficient quality health care.”[3] (emphasis ours).

Historically, black and brown women in the U.S. have been disproportionately subjected to policies that undermine both reproductive autonomy and reproductive justice. Notable historical examples include forced sterilization and other practices of the eugenics movement during the first half of the 20^th^ century.[4, 5]

Presently, examples of threats to reproductive autonomy and justice related to federal immigration enforcement in the United States are common in the media. For example, detained women have been denied access to abortion,[6] women are forced to undergo deportation while pregnant,[7] and immigrant parents have been forcibly separated from their children.[8]

Latino immigrants without citizenship status—much like the incarcerated or institutionalized women from past eugenics movements[5]—lack the same rights and privileges of free citizens. Research shows that most undocumented immigrants and their families live in perpetual fear of violence, detention, and deportation by Immigration and Customs Enforcement (ICE) and have modified their behaviors as a result.[9–12] Additionally, immigrant women with legal status are worried about enrolling in government assistance programs that can help with food assistance, prenatal care, or insurance for their children because of concerns it will inhibit their chances of renewing a visa or applying for citizenship.[11, 13] Considering the values of reproductive justice and the lived reality of Latino immigrants: What impact does immigration law enforcement targeted at certain members of their community have on reproductive autonomy among Latinos? We seek to answer this question using mixed methods data to explore how a specific immigration enforcement raid that occurred in a town in Southeast Michigan may have Latinos’ reproductive decision-making.

### Immigration law enforcement and immigrant reproductive health

The threat of immigration law enforcement can impact the health, including reproductive health, of Latino immigrants. Undocumented immigrants have been shown to have less access to health care than documented immigrants and non-immigrants.[14, 15] More recently, clinicians have been documenting that their immigrant patients who are not citizens are less willing to enroll in health insurance, food assistance programs, or travel to the clinic for fear of deportation or a negative impact on their ability to obtain a visa or citizenship status.[16, 17]

To date, there has been very limited research on the effect of immigration law enforcement on reproductive health or reproductive autonomy. Research from a community in Iowa demonstrated that infants born to Latina mothers—including both US-born Latinas and foreign-born Latinas—after a large workplace immigration raid had lower birth weights than infants born before the raid; there was no change in birthweight for White non-Latina mothers.[18] Also, recent immigration enforcement policy changes by the federal government have limited access to abortions and forcibly separated parents from their children.[6, 8]

Immigration home raids are one way in which undocumented immigrants in the interior of the U.S. are apprehended for deportation. Though no formal definition has been established, an immigration raid is generally considered to be when ICE agents enter a home or workplace and detain undocumented immigrants to process for deportation.[19] A typical home raid may involve over twenty ICE agents, often in collaboration with other law enforcement agencies, storming the location of a suspected undocumented individual in an attempt to apprehend him.[19] “Collateral” arrests, arrests of other undocumented immigrants who are present in the raided facility but not the original target of the raid, are common.[19]

Witnesses to these raids often include family members and children who could be citizens, legal residents, DACA recipients, or undocumented.[19] The phenomena disproportionately affects certain groups: 90% of arrestees/deportees are men and over 95% are Latino.[20–24]

Prior research has shown that experiencing raids or fearing immigration enforcement contribute to poor health among Latino immigrants.[9, 10, 23, 25–27] But no studies have specifically examined how immigration raids play a role in reproductive autonomy and decisions about when to have a child.

The purpose of this paper is to examine fertility intentions and reproductive autonomy within a community context in which ICE agents detain and deport undocumented community members. Specifically, we use mixed-methods to examine (a) whether an immigration raid in a community impacts Latinos’ desired timing of next pregnancy, and (b) how an immigration raid that removes several community members from their families impacts family life.

## Methods

In this study, we consider the effects of a day-long act of immigration law enforcement that culminated in the raid of a auto-repair shop in which multiple individuals were arrested and deported from a mixed-status Latino community in Michigan.[26] Our paper reports on findings from a mixed methods exploratory investigation that occurred in two parts. First, we employ a quasi-experimental design using survey data collected starting three months before and concluding two months after the raid to investigate the influence of an immigration raid on community members’ desired timing of next pregnancy. To contextualize and understand findings from this first phase, we then examined qualitative data collected in the aftermath of the raid and included in-depth interviews and participant observation with the individuals directly impacted by the raid and health/social service members serving Latinos in this setting. The University of Michigan’s Institutional Review Board approved the research.

### Context and description of the immigration raid

The county in which the study occurred has a population of about 12,000 Latinos, around 3.5% of the total county population. In the winter of 2013, a joint task force composed of agents from Immigration and Customs Enforcement (ICE) and the local sheriff’s office conducted a day long act of immigration enforcement. Originally targeting a man accused of trafficking drugs and weapons out of his automobile shop, the task force pulled over, detained, and deported multiple Latino men before raiding the automobile shop and attached apartment. Several members of a local immigration advocacy coalition were notified of the law enforcement actions and drove to the location to support those in the raided apartment. Organizers also alerted the community via a text messaging alert system, and news of the event spread on social media. By multiple accounts, including testimony of those in the raided site, advocate statements, and information obtained through the Freedom of Information Act, three women, four children under the age of five, and multiple men were in the site when it was raided. Agents broke a lock and pushed in a door, ordering women and children to the floor at gunpoint. Three days later, about 40 community members, including the women in the raided apartment and one of the study's authors, gathered at a local church. Women described their treatment and fears of the psychological repercussions on their children. Community members referenced clear feelings of racial profiling, citing that the people who were pulled over and arrested were all Latino men.

### Survey data

About three months prior to the raid, we began data collection for the *Encuesta Buenos Vecinos* (EBV; “Good Neighbor Survey”), a cross-sectional survey of Latinos—including both documented and undocumented residents—in a Michigan county to better understand their health-related needs. The researchers had no prior knowledge that the raid would occur during their data collection. Community partners were involved in the creation and review of the EBV instrument, and feedback following the pilot phase shaped implementation.

The immigration raid occurred mid-way through the survey, resulting in 325 participants who completed the EBV survey in the three months prior to the raid and 151 participants who completed the survey in the two months after the raid (total *N* = 487). No participants completed both the pre- and post-raid surveys. Because this paper focuses on reproductive autonomy, our analytic sample is a subset of Latinos who are of childbearing age and report wanting a child now or sometime in the future (*n* = 192), as further described below.

The survey was conducted in Spanish and in English, face-to-face and online, by trained members of the local Latino community, and queried such topics as health-related knowledge, access to health care, reproductive health and family planning, and neighborhood conditions. Inclusion criteria included being 18 years of age or older, self-identifying as ‘‘Hispanic or Latino/a,” and living in the county in which the survey was conducted. All participants underwent written informed consent procedures and agreed to participate in the interview.

#### Measures

We created an independent variable to capture whether participants completed the survey before or after the day of the raid. Raid timing was coded as 0 = before, 1 = after. Data were excluded from participants (*n* = 11) who completed the survey on the day of the raid or for whom the participation date could not be determined.

Our dependent variable, desired timing of next pregnancy, was assessed with the following question: “How do you feel about having a child now or sometime in the future?”, to which participants responded with one of six mutually exclusive choices: “You don’t want to have one”; “You do want to have one, less than 12 months from now”; “You do want to have one, between 12 months to less than 2 years from now”; “You do want to have one, between 2 years and 5 years from now”; “You do want to have one, 5 or more years from now”; and “You are beyond the childbearing age.”

To obtain our analytic sample, we removed participants who did not want to have any children or who self-reported being beyond the childbearing age. We recorded the variable to range 1–4 such that 1 = a desire to have a child “less than 12 months from now”, 2 = desire to have a child “12 months to less than 2 years from now,” 3 = desire to have a child “between 2 years and 5 years from now,” and 4 = desire to have a child “5 or more years from now.” This variable was treated as a four-level ordinal categorical variable in our analyses.

We controlled for the following variables due to their theoretical importance related to immigration and childbearing: age in years (continuous), sex (0 = female, 1 = male), years of education (1 = high school graduate), relationship status (0 = not in a relationship, 1 = in a relationship), years in the U.S. (continuous), the presence of children in the home (0 = no, 1 = yes), and nativity (0 = born outside of the US, 1 = born in the US).

#### Analysis

We conducted all analyses using Stata version 14. We first conducted descriptive analyses to describe the analytic sample. Then, we conducted an ordinal logistic regression model with desired timing of next pregnancy as the dependent variable and raid timing as the key independent variable, controlling for sex, age, relationship status, years in the country, the presence of children in the household, and nativity. We first ran bivariate ordinal logistics regression models with the dependent variable and each other variable. Then, given that each of our control variables is theoretically relevant to our main research question, we ran a multivariable ordinal logistic regression model to examine the effect of raid timing on desired timing of next pregnancy, controlling for socio-demographic factors.

### Qualitative data collection and analysis

In order to contextualize the findings from the quantitative analyses, we considered field notes and interviews from our qualitative research after the raid which had the primary goal of better understanding the impacts of the raid on the community in which it occurred. The interviews were not originally intended to specifically explore reproductive justice and autonomy, but the analyses undertaken for this paper examined this issue by assessing how the impacts people described were related to child-rearing, families, and/or fertility decision-making. More extensive details on the qualitative methods used are available elsewhere.[28]

The qualitative portion of the study was informed by principles from grounded theory[29, 30] and consisted of in-depth interviews with 21 individuals and approximately 260 hours of structured participant observation in a range of locations relevant to the mixed-status community in which the raid occurred. Interviews and participant observation were conducted by the second author, a Latino male doctoral student in public health at the time of the data collection. He had extensive prior training and experience in qualitative data collection and participant observation. These formal data collection efforts were complemented by extensive work of study team members in the Latino community over the past decade (including prior to the start of the current study). All of these study procedures were approved by the University of Michigan Institutional Review Board.

Participants were recruited through snowball sampling. Initially the interviewer was connected with participants through connections with a local community-based organization serving Latino families and then relied on referrals to other participants (particularly those affected by the raid). Inclusion criteria for participants included being age 18 or over and living in Washtenaw County at the time of the raid. Interviews focused on two sub-groups: interviews with family members directly involved in the raid (n = 10) and representatives from social service agencies serving Latino immigrant clients in the region (n = 11). Interviews were conducted in a private location selected by the participant. All participants underwent written informed consent procedures and agreed to participate in the interview.

Interviews were digitally recorded and transcribed in their original language by a trained transcriptionist. Then, after the second author compared transcriptions with field notes from both the interviews and participant observation, each interview was extensively memoed and a codebook was developed with both inductive and deductive codes.[29, 31] Transcripts were then coded using NVivo. For the current analysis, we focused on codes such as “economic effects,” “effects on children,” “individual level health,” and “interpersonal relationships” in order to better contextualize aspects of reproductive decision-making, reproductive justice and autonomy. We examined these codes for references to raising and providing for children, childbirth, and family. Additionally, we connected these quotes to observations from the community in fieldnotes to develop our understanding of desired timing of next pregnancy in this context. In the results section, we describe the salient findings related to desired timing of next pregnancy and immigration enforcement to contextualize the quantitative findings.

## Results

### Survey findings

Of the 192 participants in our analytic sample, 54% were women. The average age was 27.6 years (*SD* = 6.7). Seventy-four percent were born abroad and their average time in the US was 14 years (*SD* = 8.8). The average years of education was 12.8 years (*SD = 4*.*0*). More than half (58%) of participants were in a relationship and 42% had children at home. Among our analytic sample of Latinos who wanted to have more children, 16% wanted to have a child in the next 12 months, 13% wanted to have a child in the next 12 months to 2 years, 34% wanted to have a child between 2 and 5 years from now, and 38% wanted to have a child more than 5 years from now. See Table 1 for full descriptive results.

**Table 1:** Sample characteristics of participants and comparison between before raid/after raid participants, *n* = 192.

Measure	Full sample (*n* = 192)	Before raid (*n* = 120)	After raid (*n* = 72)	X^2^/*t* value, *p* value
Women (%)	54.2	58.3	47.2	2.2, .135
Age, years (mean, SD)	27.6 (6.7)	28.0	27.0	1.0, .336
Time in US, years (mean, SD)	14.1 (8.8)	13.0	15.9	-2.2, .027
Education, years (mean, SD)	12.8 (4.0)	12.7	13.0	-0.6, .556
Born abroad (% yes)	74.1	79.7	65.3	4.7, .030
Children in home (% yes)	41.9	44.2	38.0	0.7, .406
In a relationship (% yes)	58.1	55.5	62.5	0.9, .339
Desired timing of next pregnancy (% yes)				12.2, .007
Child in < 12 months	16.2	20.0	9.7	
Child in 12 months to 2 years	12.5	16.7	5.6	
Child in 2 to 5 years	33.9	33.3	34.7	
Child in 5 or more years	37.5	30.0	50.0	

Our data show that, on average, participants who completed the survey after the raid preferred to delay childbearing compared to those who completed the survey before the raid (See Table 2 for bivariate and multivariate results). Results from our bivariate ordinal logistic regression showed that participants were more likely to prefer waiting a longer period until their next pregnancy if they were interviewed after the raid (Odds Ratio [OR]: 2.54, 95% Confidence Interval [CI]: 1.46–4.41) and more likely to prefer a shorter waiting period if they were older in age (OR: 087, 95% CI: 0.79–0.87), in a relationship (OR: 0.34, 95% CI: 0.20–0.59), or already had children in the house (OR: 0.25, 95% CI: 0.14–0.44). Results of the multivariable ordinal logistic regression model show that raid timing (Adjusted OR: 2.13, 95% CI: 1.13–3.99) and age (Adjusted OR: 0.84, 95% CI: 0.80–0.89) remained significantly associated with desired timing of next pregnancy while holding all other variables constant. Specifically, among Latinos who wanted to have another child, people interviewed after the raid had 2.13 times the odds of wanting to wait a longer period of time until their next pregnancy compared to people interviewed before the raid. For age, there was an inverse relationship such that for each year increase in age was associated with 16% lower odds of wanting to wait a longer period of time until their next pregnancy (holding all other variables constant).

**Table 2:** Multivariable ordinal logistic regression analysis of raid timing and covariates on desired timing of next pregnancy (*n* = 181).

	OR	95% CI	*p*	Adjusted OR	95% CI	*p*
Raid timing (1 = after)	2.54	1.46–4.41	0.001	2.13	1.13–3.99	0.019
Age	0.83	0.79–0.87	<0.001	0.84	0.80–0.89	<0.001
Sex (1 = male)	1.28	0.76–2.15	0.349	1.29	0.72–2.33	0.395
Education (1 = HS graduate)	1.28	0.76–2.15	0.359	0.92	0.45–1.88	0.812
Relationship status (1 = in a relationship)	0.34	0.20–0.59	<0.001	0.58	0.30–1.11	0.101
Years in U.S.	1.01	0.98–1.04	0.631	1.01	0.97–1.06	0.533
Children in house (1 = yes)	0.25	0.14–0.44	<0.001	0.53	0.25–1.12	0.096
Nativity (1 = born in the US)	2.55	1.38–4.69	0.003	1.00	0.41–2.46	0.998

### Qualitative findings

Results of our multivariate analyses suggest that community members impacted by an immigration home raid may be more likely to want to delay childbearing.

#### Financial effect of the raid

Detention and deportations that occurred because of the raid prompted the removal of financial providers and thrust families into sudden poverty or exacerbated the poverty many already experienced. Families frequently mentioned that they were unsure of how children would be fed or clothed, or how they would provide their infants with the diapers they needed daily.

Santiago, the original target of the raid, was arrested as he drove away from the targeted facility and was deported soon after. When asked about the hardest moment of the experience, he referenced the dire financial straits into which his family had been forced:

*It’s hard*, *it’s so hard because my sister*, *my nephew and nieces*, *my wife*, *my children*, *all were under my care*, *as I took care of everything that they needed… What I earned I earned for diapers*, *milk*, *food for the house*. *Everything for my nephew and nieces*, *my sister*, *my wife*, *my children… I would ask myself*, *‘Now who is going to help them? Who is going to give them what is needed?’*

Faced with the lack of her primary provider, his wife found it too challenging to raise her children, and eventually left the U.S. to be with her husband, leaving most of their material possessions behind in Michigan. Other family members directly impacted by the raid also mentioned that the raid caused an extreme financial strain on the family because of legal fees and the loss of income.

#### Psychological effect of the raid on children

Children were also traumatized following the raid. For those children in the raided apartment, the psychological impacts of being in a building stormed by heavily armed officers were evident in behavior changes in the days and months that followed. Yet, children who were not in the raided facility were also impacted, as they began to fear that every time their parents drove away they may never return.

Erlinda, Santiago’s sister, described the words of her son who, was about 4 years old at the time of the raid: said,

*[My son] says*, *‘It scares me when you leave the house*, *when you drive*, *because I don’t want the police to take you like they took my dad.’ He says*, *‘or like they took my uncle Santiago.’*

Ignacio, also detained on the day of the raid, described his children’s perpetual fear of police:

*Every time somebody knocked the front door*, *or they see a police car*, *even if it’s a mile away that was enough to just freak the hell out*, *like they just anxious…. they [weren’t] the same. Their reaction was completely like another person because they afraid*, *they insecure*, *they angry*, *they just for what they did to me*.

This trauma and fear that children experienced was commonly reported among family member impacted by this raid. Additionally, our fieldwork and interviews with advocates made clear that it is extremely common for the children of undocumented immigrants to experience this fear given that they have heard stories of their classmates’ parents being detained or deported.

#### Community fear after the raid

According to our participant observations and interviews, news of the raid spread rapidly, and many throughout the community knew those involved or were familiar with the area. Thus, the presence of ICE—and, to some extent, the police—signaled the potential for the catastrophic: you or a member of your family could be forcibly removed. Karina, a social service agency representative who was involved in working with the community after the raid described the impact ICE presence can have on parents and children:

*Most recently we had ICE Fugitive Ops stopping parents coming out of [an] elementary school. And just scaring those children to death*, *not to mention the parents. And the school went into a lock down mode. So we’re talking about [ICE] having the same effect as having a shooter in the school*.

Karina has extensive experience working with mixed-status families and she describes how the negative effect of ICE presence—and the threat of forced removal of a family member—on mixed-status families could be similar to other traumatic events that could happen to children.

The immigration enforcement actions kindled a fear in community members of *all* government services, not just ICE or the police. Members of social services agencies reported that the use of social services, including those to which children are entitled, decreased due to fear of immigration enforcement. As one representative of a social service agency describes:

*But those women and children were like homeless … unless the community takes them in and helps them*, *there is really nothing for them*, *nothing. The kids can get food stamps but the mother is probably terrified. Do you think she is going to go out to [the Department of Health and Human Services] and apply for food stamps? She’s probably afraid the minute she walks in there*, *they’ll call immigration. … And that’s a shame*, *cause the kids*, *kids are born here*, *they have a right to eat*.

Not every community member had direct contact with ICE or the police as did Ernesto and Ignacio but many Latino immigrants have a need for government services. Given the severe consequences for a family member being detained or deported, many families chose to avoid government programs or services they were eligible for. Even though not every community member came in direct contact with ICE or the police, news of the raid spread rapidly, and many Latinos—particularly those in mixed-status families—exercised extreme caution to help increase the odds that their family would not experience the same catastrophe.

## Discussion

We found that, among Latinos of childbearing age who wanted to have more children, individuals who had recently been exposed to an immigration home raid in their community wanted to delay their next child longer than individuals who had not been recently exposed to an immigration raid. Further, we find support from our qualitative research that this delay post-raid may be explained by the pervasive anxiety related to having a family member forcibly removed and the likely negative financial and mental health impacts on those left behind. In this section, we connect our findings to previous literature and highlight the importance of these findings for the current immigration enforcement climate.

Our findings on the impact of a specific raid echo research by Novak, Geronimus, & Martinez-Cardoso.[18] Their research demonstrated that infants born to Latina mothers in Postville, Iowa after a large workplace raid targeting Latino workers took place had lower birth weights than infants born before the raid; at the same time, there was no change in birth weight for white non-Latina mothers.[18] The explanation for this difference is that the immigration raid was a racialized stressor that impacted birth weight for Latina women.[18] Together with our findings, these results suggest that immigration raids play a role in reproductive health, including an individual’s decision to have children. More mixed methods research is needed to further delineate which reproductive health outcomes are affected by immigration enforcement and the mechanisms that drive the relationships.

The findings from our qualitative interviews contribute to a growing body of literature that documents the fear experienced by families that are under the constant threat of deportation. Recent papers by Doering-White et al. and Lopez et al. analyzed data collected from in-depth interviews with undocumented mothers in the Midwest who have had someone close to them deported.[23, 32] Delva and colleagues interviewed 20 children from mixed-status families where at least one parent was undocumented and found that many of those children experienced poor mental health.[33] Like findings from our study, these papers similarly highlight the negative psychosocial and economic effects on the women and their children that either experience deportation in their family or live in a mixed-status community where family members are removed by immigration enforcement actions.

Our findings are particularly notable given the recent increase in anti-immigrant rhetoric and changes in immigration enforcement since the 2016 U.S. presidential election. While enforcement of immigration laws has always been part of federal policy, the federal government has recently broadened the criteria for immigrants it targets for raids and deportation. Since President Trump took office, he has expanded the number of Immigration and Customs Enforcement (ICE) officers making arrests and there has been a 40% increase in detentions of undocumented immigrants.[34] Unlike immigrant detention and deportation under the Obama administration which focused primarily on ‘serious criminals,’ the standard practice under the Trump administration is to target any immigrant who has broken the law (including entering the U.S. unlawfully) and to round up any other immigrants that are nearby at the time of arrest.[34] As part of these new changes, the federal government has reinstated large-scale workplace raids that detain hundreds of immigrants, last utilized under the Clinton and Bush administrations. The government also has chosen to forcibly separate parents from their children when they cross the border.[8]

Given these changes to immigration policies, we hypothesize that the fear and anxiety of an immigration raid is now a common sentiment among individuals living in mixed-status immigrant communities. Indeed, several contemporaneous news reports and emerging research highlight that this is the case.[11, 35] More research is needed to examine whether immigrants—particularly Latino immigrants since they are the target of most raids—have changed their fertility intentions and other dimensions of reproductive autonomy after the 2016 election.

Another potential concern for reproductive justice and autonomy is that these expanded immigration detention efforts have resulted in some undocumented immigrants and family members being unwilling to risk deportation to seek health and social services.[16] Contributing to this concern, a draft executive order by President Trump threatens to revoke current ICE policies that protect immigrants from being detained or deported at health care facilities.[36] Recent research also highlights that some women are avoiding prenatal care or WIC services due to fears of immigration enforcement.[11] Women and men may be intending to delay childbearing while simultaneously having less access to family planning services, contraceptives, or abortions to actually fulfill their fertility intentions.

Given increasing immigration enforcement that separates families, our findings point to the need for family-friendly immigration policies to expand reproductive autonomy and justice for immigrants.[37] Immigration policy reform could include a provision for an affordable path to citizenship for undocumented parents that could eliminate the fear of forced removal from these families. This policy solution would likely improve outcomes—and reproductive autonomy—for millions of U.S. family members.

### Limitations

Our findings should be viewed in light of several limitations. First, our cross-sectional survey data do not allow us to examine individual changes in desired timing of next pregnancy before and after a home raid. Instead, given our (unintended) quasi-experimental design, we can compare members of a population before a raid with those responding after a raid. Future longitudinal research could assess this more directly. Second, we are unable to distinguish the potential different effect between documented Latinos and undocumented Latinos. We would hypothesize that the effect would be stronger for undocumented Latinos. Third, we have a relatively small sample of participants in the ‘post-raid’ category; a larger sample size would allow for more statistical power for more confidence in the effect size. Fourth, our qualitative data collection was not intended to explicitly explore fertility intentions and focused primarily on those people impacted directly by the raid. Thus, it does not represent the entire community. Thus, using our qualitative findings to contextualize the survey findings may not include all relevant perspectives and feelings of community members. Future research should use qualitative methods to explore how fertility intentions change when a immigration raid occurs nearby (but does not directly impact the individual). Finally, the immigration raid occurred in 2013 during a very different immigration enforcement regime. Since immigration enforcement has increased since early 2017,[34] we would expect the pervasive fear described in the qualitative findings to be even more prevalent today. Nonetheless, further research is needed to explore these dynamics in the current immigration enforcement climate.

## Conclusions

When the threat of deportation looms over families, their decisions are affected and their autonomy is restricted. Our findings point to the need for family-friendly immigration policies that prioritize keeping families safe and whole.[37] Improving reproductive autonomy and health within mixed-status families may be improved with small local-level interventions but will ultimately require federal immigration reform.
